# Temporal and structural sensitivities of major biomarkers for detecting neuropathology after traumatic brain injury in the mouse

**DOI:** 10.3389/fnins.2024.1339262

**Published:** 2024-01-30

**Authors:** Guoxiang Xiong, Ian Jean, Anthony M. Farrugia, Hannah Metheny, Brian N. Johnson, Noam A. Cohen, Akiva S. Cohen

**Affiliations:** ^1^Department of Anesthesiology and Critical Care Medicine, The Children’s Hospital of Philadelphia, Philadelphia, PA, United States; ^2^Philadelphia Veterans Affairs Medical Center, Philadelphia, PA, United States; ^3^Department of Otorhinolaryngology−Head and Neck Surgery, Philadelphia, PA, United States; ^4^Department of Anesthesiology and Critical Care Medicine, Perelman School of Medicine, University of Pennsylvania, Philadelphia, PA, United States

**Keywords:** brain trauma, varicosity, axonal truncation, dendritic arbor, cell debris, β-Amyloid, silver staining

## Abstract

Traumatic brain injury (TBI) is a leading cause of morbidity and mortality, especially in teenagers to young adults. In recent decades, different biomarkers and/or staining protocols have been employed to evaluate the post-injury development of pathological structures, but they have produced many contradictory findings. Since correctly identifying the underlying neuroanatomical changes is critical to advancing TBI research, we compared three commonly used markers for their ability to detect TBI pathological structures: Fluoro-Jade C, the rabbit monoclonal antibody Y188 against amyloid precursor protein and the NeuroSilver kit were used to stain adjacent slices from naïve or injured mouse brains harvested at different time points from 30 min to 3 months after lateral fluid percussion injury. Although not all pathological structures were stained by all markers at all time points, we found damaged neurons and deformed dendrites in gray matter, punctate and perivascular structures in white matter, and axonal blebs and Wallerian degeneration in both gray and white matter. The present study demonstrates the temporal and structural sensitivities of the three biomarkers: each marker is highly effective for a set of pathological structures, each of which in turn emerges at a particular time point. Furthermore, the different biomarkers showed different abilities at detecting identical types of pathological structures. In contrast to previous studies that have used a single biomarker at a single time range, the present report strongly recommends that a combination of different biomarkers should be adopted and different time points need to be checked when assessing neuropathology after TBI.

## Introduction

Traumatic brain injury (TBI) is a leading cause of morbidity and mortality, especially in teenagers to young adults (Coronado et al., 2011). TBI results in neurodegeneration in the cerebral cortex, hippocampus and other key structures, which often appears as neuronal loss (Witgen et al., 2005), oligodendrocyte death and diffuse axonal injury (Adams et al., 1989; Povlishock, 1992; Flygt et al., 2016; Xiong et al., 2023). TBI-induced neurodegeneration may subsequently evolve into chronic dementia as in Alzheimer’s or Parkinson’s diseases (Johnson et al., 2013). In studies focused on the early stages of TBI, many different biomarkers and/or staining protocols have been developed in recent decades, and many contradictory findings have been reported. Some of these conflicting results may be due to differences in technique. For instance, neuronal death after TBI has been documented (Yang et al., 2015, 2020; Koch et al., 2020) using histological staining with Fluoro-Jade dyes (Schmued et al., 1997, 2005; Schmued and Hopkins, 2000). However, these dyes are not specific for dying or dead cells because they can also stain normal and/or activated astrocytes (Colombo and Puissant, 2002; Chidlow et al., 2009; Xiong et al., 2023). Immunoperoxidase-ABC staining for the N-terminal of amyloid precursor protein (APP) with a mouse monoclonal antibody (Clone 22C11) has shown diffuse axonal injury as varicosities or spheroids in white matter (WM) bundles in TBI patients (Gentleman et al., 1993, 1995; Sherriff et al., 1994a,b). This protocol has even been recommended as the “Gold standard” for detecting axonal damage after TBI (Johnson et al., 2013, 2016). However, 22C11 may not be an ideal marker for APP (Guo et al., 2012; Del Turco et al., 2016) and we could not detect varicosities or spheroids in injured mice when using immunofluorescent staining (as opposed to immunoperoxidase-ABC protocol) with 22C11 (Xiong et al., 2023). By contrast, C-terminal antibodies against APP (Clones Y188, CT695 and CT-20) can be used to detect axonal blebs (or truncation of axons) in injured mice (Stone et al., 2000; Singleton et al., 2002; Wang et al., 2011; Flygt et al., 2013; Xiong et al., 2023), in a time-dependent manner (Xiong et al., 2023). With regard to silver staining, whereas the Palmgren protocol could not clearly show neurodegeneration in TBI patients (Gentleman et al., 1995), the Gallyas protocol (Gallyas et al., 1980) has succeeded in revealing degenerated neurons and/or neuropil in experimental rodents (Koliatsos et al., 2011; Haber et al., 2017; Borniger et al., 2018; Xiong et al., 2023). In order to compare these major biomarkers for their ability to detect specific degenerative structures, we performed Fluoro-Jade C (FJC) staining, Y188 immunostaining and NeuroSilver staining in adjacent brain slices from naïve or injured mice subjected to lateral fluid percussion injury (lFPI). Since axonal blebs undergo morphological changes as the pathology progresses (Xiong et al., 2023), we paid a special attention to different time points after TBI in the present study.

## Materials and methods

Eight-week-old male C57/Bl6 mice from Jackson Laboratory (Bar Harbor, ME) were used. The procedures and protocols for all animal studies were approved by Institutional Animal Care and Use Committees of Children’s Hospital of Philadelphia and University of Pennsylvania, in accordance with international guidelines on the ethical use of animals (National Research Council, 1996). To make TBI mice, we followed lateral fluid percussion injury (lFPI) protocol as reported previously (Schwarzbach et al., 2006; Xiong et al., 2014), with a modified impact (2.6–2.7 atm peak pressure) for moderate lFPI (Xiong et al., 2023). Injured mice with gross cortical damage visible to the naked eye were excluded from the present study. Furthermore, cases showing no FJC-stained cortical and hippocampal neurons were excluded from lFPI groups. Sham mice received craniectomy, without fluid percussion applied.

After the respective times for survival (as indicated in the text), lFPI and age-matched naive or sham mice were deeply anesthetized with 5% chloral hydrate and perfused with 4% paraformaldehyde (PFA) in 0.1 M phosphate buffer (PB) at room temperature (RT). Brains were removed and post-fixed in the same fixative for 90 min at RT. Frontal slices were cut in PB at 50 μm of thickness with a Leica VT 1000s vibratome (Leica Microsystems Inc., Buffalo Grove, IL). To minimize the number of animals used, 6 series of slices from each brain were collected in PB for staining with all three markers, with an interval of 300 μm between two successive slices in each series (Xiong et al., 2012, 2023). If slices needed to be stored for longer periods of time, e.g., 2 weeks to several months, we added sodium azide at 0.1% into the storage medium. This strategy allowed us to minimize the number of animals needed, because we could use in the current study some of the tissue (including naïve, sham and injured) collected for the previous study. For each time point, at least three pairs of mice (naïve and lFPI) were used for staining. When a negative result was acquired, we added another pair of mice to confirm the negative finding.

FJC staining (Schmued et al., 2005) was performed prior to immuno- or silver staining. Vibratome slices were fixed again in 4% PFA at 4 °C for 24 hr. Treating the slices with 0.06% potassium permanganate prior to staining with 0.0004% FJC (Histo-Chem, Inc., Jefferson, AR), was perfromed as described previously (Xiong et al., 2023). To reveal APP immunoreactivity in the brain, we performed free floating immunoperoxidase-ABC staining (Xiong and Matsushita, 2000) with Y188 (Xiong et al., 2023). To show the interference in ABC staining caused by endogenous biotin (Bhattacharjee et al., 1997; McKay et al., 2008) that is present in the brain (LeVine and Macklin, 1988; Wang and Pevsner, 1999; McKay et al., 2004), we performed direct staining in adjacent slices with Avidin-HRP (Vector Labs, Burlingame, CA). After peroxidase blocking with H_2_O_2_ and raising permeability with Triton X-100, the slices were incubated with Avidin-HRP (1:100) for 60 min before visualization with DAB-H_2_O_2_ for 15 min (Xiong et al., 2023). One series of vibratome slices was further fixed in 4% PFA at 4 °C for 7 d, prior to Gallyas staining with NeuroSilver Kit (PK301; FD NeuroTechnologies Inc., Columbia, MD). This silver staining kit was designed to selectively stain degenerated neurons and/or neuropil (Koliatsos et al., 2011; Xiong et al., 2023), based on the argyrophilic feature of these deformed structures (Gallyas et al., 1980). The free-floating protocol optimized by the manufacturer was strictly followed, with special precautions to avoid sodium and ethanol (Xiong et al., 2023).

We also performed Golgi staining in naive brains, in order to compare the morphology of FJC-stained neurons to normal ones. Mice were perfused with 10% neutral formalin and whole brains were post-fixed overnight at 4°C. The brains were then treated in dark with a mixture of 1% osmium tetroxide and 2.5% potassium dichromate (1:100 in volume) for 5 d. After rinsing with 0.75% silver nitrate until clear, the brains were immersed in silver nitrate solution for 48 hr. Golgi-stained brains were sectioned at 150 μm in 95% ethanol with a Leica VT1000S vibratome. Slices were collected in 95% ethanol and dehydrated twice with 100% ethanol for 3 min. They were then cleared with xylene twice for 5 min, before being mounted on a precleaned glass slide and coverslipped with Permount.

Previous data (Xiong et al., 2023) were also reviewed and taken into consideration when drawing the summary (Table 1), since all 3 markers were used in both studies.

**TABLE 1:** Staining efficiency and time window of three major biomarkers for detecting pathology after TBI.

Positive staining	Biomarkers Comparative efficiency (Time point)		
	**FJC**	**Y188**	**NeuroSilver kit**
Neuronal cell bodies	+++ (1 hr-7 d)	+ (4–5 d)	++ (18 hr-7 d)
Beaded deformation of dendrites	+++ (6 hr-7 d)	−	+++ (18 hr-7 d)
Axonal blebs	−	++ (1 hr-5 d)	+++ (0.5 hr-2 d)
Wallerian degeneration	++ (5 d-1 m)	−	+++ (5 d-3 m)
Axonal bulbs	±	−	+++ (5 d-1 m)
Patches of puncta in white matter	−	+++ (3 hr-7 d)	−
Perivascular aggregates in white matter	−	+++ (1 hr-7 d)	−
“Non-specific staining”	Astrocyte, basal body and pial fiber	Endogenous biotin (Immunoperoxidase- ABC protocol)	Oligodendrocyte, pial fiber and ventricular bank

Comparative efficacy scaled as “+ to +++” and time points (hr, d, or m: h, day or month, respectively, after lFPI) when positive staining emerges. Negative staining marked as “–”. “Nonspecific staining” in naïve mice also listed.

## Results

### FJC staining after lFPI

**FIGURE 1:**
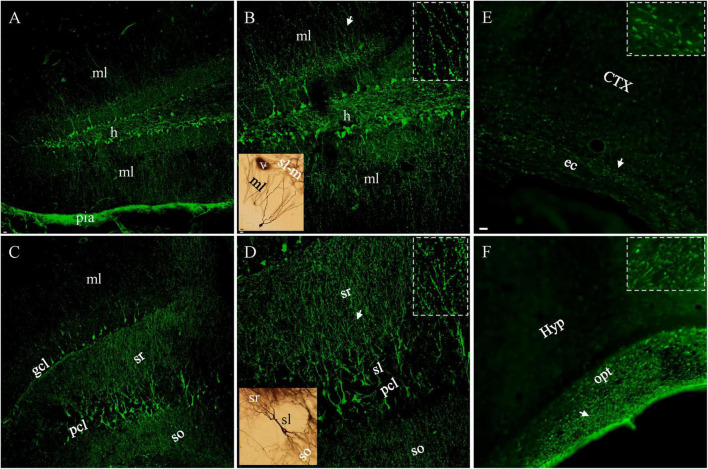
FJC-stained neuronal cell bodies and dendrites 10 h and Wallerian degeneration (of axons) 7 d after lFPI. **(A)** Dentate granule cells were intensely stained in both supra- and infrapyramidal blades, with obvious staining in the hilus and both molecular layers. Note the most intense staining in pia mater. **(B)** Granule cells and dendrites under higher magnification. Beaded distal dendrites (*arow*) highlighted in (*Inset*). **(C)** Pyramidal neurons in area CA3c and apical (sr) or basal (so) dendrites stained with FJC. **(D)** FJC-stained pyramidal cells and dendrites under higher magnification. More beaded staining all over dendritic layers in CA3 (*inset*) than that in dentate gyrus panel [**(B)**, *inset*], except for proximal dendrites (sl). A Golgi-stained normal granule or pyramidal cell (black, *insets in brown color*) was shown for a comparison to FJC-stained counterpart cells. **(E,F)** FJC-stained Wallerian degeneration in external capsule (ec) and optical tract (opt), with indicated areas (*arrows*) highlighted (*insets*). CTX, cortex; gcl, pcl, granule and pyramidal cell layer; h, hilus; Hyp, hypothalamus; ml, molecular layer; sl, so and sr, stratum lucidum, oriens and radiatum. Scale bars: 20 μm in panels **(A,C)**; 10 μm in [**(B, D)** and *insets for Golgi-stained cells*]; 5 μm in **(E,F)**, with 2 μm for *insets*.

We performed FJC staining after lFPI to evaluate its capacity to assess neuronal damage (Yang et al., 2015, 2020; Xiong et al., 2023). Neuronal staining was never seen in control (naïve) animals, except for astrocytic staining (Xiong et al., 2023). In lFPI mice, intensely stained neuron cell bodies, together with the proximal dendrites could be identified in the cerebral cortex as early as 1 h after injury. FJC-stained neurons were also seen in the dentate gyrus and area CA3 of the hippocampus (Figures 1A–D). Another typical pattern of FJC staining was beaded deformation along the dendritic trees of these positive cortical and hippocampal neurons after lFPI. This characteristic pathology emerged from 6 hr after injury and reached a peak density at 10 h after injury (Figures 1A–D), similar to that revealed in cultured neurons (Monneriea et al., 2010) and Drosophila (Tao and Rolls, 2011). The somata of FJC-stained cells appeared to be intact (Figures 1B, D), similar to those of Golgi-stained neurons in naïve mice (black structures in brown background insets). The number of FJC-stained neuronal cell bodies reached a peak density at 3 hr after lFPI, was dramatically decreased after 2 d and could rarely be found at 7 d after lFPI. However, beaded deformation of dendritic trees remained clearly identifiable at this later time point. FJC staining also showed Wallerian degeneration of axons (Figures 1E,F), appearing as parallel dotted-lines in almost all white matter bundles from 5–7 d after injury (insets), and persisted up to 1 month after lFPI.

### Immunoperoxidase-ABC staining for APP with Y188 after lFPI

**FIGURE 2:**
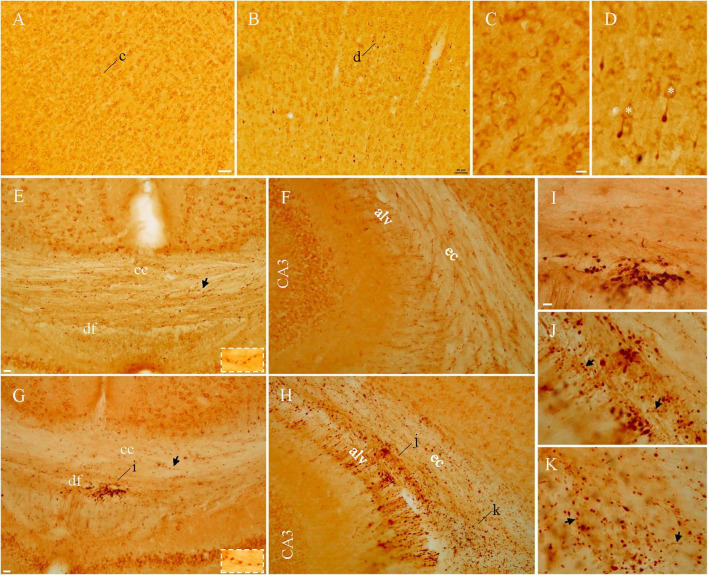
Axonal blebs and puncta identified in injured mice with Y188 immunoperoxidase-ABC staining. In addition to basal staining in normal neurons **(A)**, intensely stained axonal blebs could be found in the cortex from injured mice **(B)**. Under higher magnification, basal staining **(C)** was seen to encircle cell membrane and axonal blebs **(D)** appeared to be dead ends of the remaining axonal segments that were connecting to basal-stained parent cell bodies (*asterisks*). Basal staining was also seen in white matter bundles **(E,F)** in naïve mice, whereas large clusters of intensely stained puncta could be seen in injured mice **(G,H)**, indicated by **(i–k)** and highlighted in panels **(I–K)**. Note that corpus callosum from injured mice was devoid of intensely stained puncta **(G)**. Varicosity-like arrangements could be seen in both naïve and injured mice panels [**(E,G,J,K)** arrows and/or insets]. alv, alveus; cc, corpus callosum; df, dorsal fornix; ec, external capsule. Scale bars: 50 μm in panels **(A,B)**; 10 μm in panels **(C, D,I–K)**; 20 μm in panels **(E–H)**.

Y188 staining in naive mice revealed immunoreactivity evenly distributed in gray matter areas including the cortex as shown in Figure 2A, reflecting the role of APP in neuronal morphology, spine density, synaptic plasticity and learning/memory (Hick et al., 2015; Müller et al., 2017). We refer to this staining pattern in normal brains as “basal staining” (Xiong et al., 2023), to distinguish it from the pathological staining found in injured brains. Note that unlike Y188, 22C11 immunoperoxidase-ABC staining had shown negative results in control tissue (Gentleman et al., 1993, 1995; Sherriff et al., 1994a,b). As early as 1 hr after lFPI we encountered axonal blebs among basal-stained neuronal cell bodies in gray matter areas including the cortex (Figure 2B), hippocampus and nucleus caudatus-putamen. Under high magnification, basal staining appeared to be membrane staining encircling the cytoplasm of neurons (Figure 2C) and axonal blebs were visible as intensely stained spheres at the distal end of a remaining axonal segment connecting to the parent cell body (Figure 2D), similar to the morphology of axonal truncation demonstrated previously (Stone et al., 2000; Singleton et al., 2002; Wang et al., 2011; Flygt et al., 2013). Another form of pathology detected by Y188 in gray matter was condensed neuronal cell bodies at 4–5 days after lFPI, as reported previously (Xiong et al., 2023). Basal staining was also seen in white matter bundles including the corpus callosum, dorsal fornix (Figure 2E), alveus and external capsule (Figure 2F) from naïve mice. From 3 hr after lFPI Y188 staining showed pathological structures in white matter bundles, which would form large patches of intensely stained puncta (Figures 2G–K). Among patches of these small-sized puncta, we also found axonal blebs. The last type of Y188-detectable pathology in white matter bundles was aggregates of intensely stained perivascular structures, which started to emerge at 1 hr after lFPI along ventricular banks. We refer to axonal blebs, condensed cell bodies, patches of puncta and perivascular structures as “pathological staining”, as they were never seen in naïve mice (Xiong et al., 2023). Such Y188-stained pathology decreased dramatically by 7 d after lFPI and was only rarely observed at 2 weeks following FPI. Both basal staining (Figures 2E,G) and pathological staining (Figures 2I–K) in white matter bundles could exhibit varicosity-like arrangement (*arrows and insets*). The above Y188 immunoperoxidase-ABC staining pattern in naïve and injured mice is consistent with the Y188 immunofluorescent staining shown in our previous report (Xiong et al., 2023).

Y188-stained axonal terminals could be identified in layers I-II of cortex at the site of craniectomy after sham surgeries, together with a few axonal blebs in layer III to VI. Aggregates of perivascular structures could also be found in gray matter of the cortex from sham animals. These findings are highly consistent with our previous study (Xiong et al., 2023), and supportive of previous reports demonstrating pathological, behavioral and neurochemical alterations from the craniotomy alone (Cole et al., 2010; Wu et al., 2013). Since sham surgery-induced pathology is a potential confound in any study using an open skull injury model, we used naïve animals as negative controls in both the previous (Xiong et al., 2023) and present studies.

### Gallyas staining with neurosilver after lFPI

**FIGURE 3:**
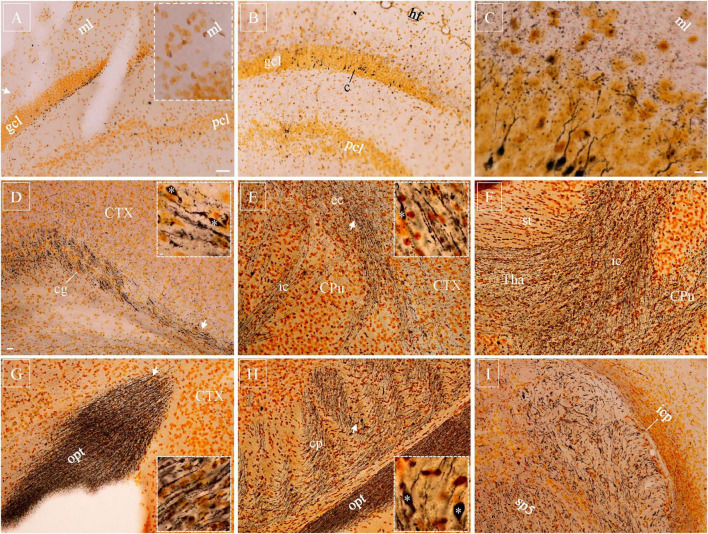
Silver-stained axonal blebs, neuronal cell bodies with dendrites, and Wallerian degeneration with axonal bulbs after injury. **(A)** Silver-stained axonal blebs were distributed in subgranular zone of the dentate gyrus (suprapyramidal blade) 10 h after lFPI, without dendritic staining in the molecular layer (*inset*). **(B)** Number of axonal blebs enormously decreased at 18 h and some cell bodies got intensely stained, together with dendrite shafts. **(C)** At higher magnification, distal part of the dendritic tree exhibited beaded appearance in the molecular layer. **(D–I)** Silver-stained Wallerian degeneration distributed diffusively in white matter bundles 7d after lFPI, as exemplified in cingulum **(D)**, internal/external capsule **(E,F)**, optical tract **(G)**, cerebral peduncle **(H)**, and spinal trigeminal tract and inferior cerebellar peduncle **(I)**. Representative subregions (*arrows*) are highlighted (*insets*), together with some axonal bulbs (*asterisks*). CPu, nucleus caudatus-putamen; sp5, spinal trigeminal tract; Tha, thalamus. Scale bars: 50 μm in panels **(A,B)**; 10 μm in **(C)**; 20 μm in **(D–I)**, with 2 μm in (*insets*).

Silver-stained axonal blebs could be clearly seen in the cortex, hippocampus and nucleus caudatus-putamen as early as 30 min after lFPI. They were regular spheres in shape and much smaller than neuronal cell bodies which had only background staining (Xiong et al., 2023). Unlike Y188 staining, silver-stained axonal blebs did not carry connecting axonal segments. In the hippocampus, silver-stained blebs were distributed exclusively in the subgranular zone of the dentate gyrus (Figure 3A) and infrapyramidal zone of area CA3. At 10 hr after lFPI, cellular and/or axonal debris started to appear around axonal blebs, indicating decomposition of the injured neurons and/or axons as suggested previously (Xiong et al., 2023). From 18 hr after lFPI, beaded deformation could be seen along dendritic trees in the cortex, hippocampal dentate gyrus and area CA3, together with some heavily stained cell bodies and proximal dendritic shafts in principal neuron layers (Figures 3B,C). Silver-stained cell bodies and deformed dendritic arbors reached their peak density 48 hr after lFPI and remained identifiable up to 1 w post-injury.

Diffuse Wallerian degeneration of axons (Figures 3D–I, and *insets*) could be seen 5–7 d after lFPI, exhibiting as parallel dotted lines in most white matter bundles. A few axonal bulbs (Figure 3, *asterisks*) were identifiable among these Wallerian degenerated axons. Wallerian degeneration could also be spotted in gray matter, together with a few condensed cell bodies and debris. Dotted Wallerian degeneration remained clearly detectable up to 1 month after lFPI, implying persistent and/or ongoing axonal damage after TBI. Three months after lFPI, a very limited number of axons could be stained in white matter bundles. At this very late timepoint, however, NeuroSilver staining usually showed intact axons, having very little dotted morphology.

Cell bodies, beaded dendrites and axonal blebs stained by one or more biomarkers were seen predominantly ipsilateral to lFPI, except for a few in the contralateral dentate gyrus (Xiong et al., 2023). Wallerian degeneration was diffusively distributed on both sides of the brain. Y188-stained patches of puncta and perivascular staining in white matter were exclusively ipsilateral.

### “Non-specific” staining by the three biomarkers of interest in naïve mice

Together with the characteristic and specific pathological staining patterns demonstrated above, all three biomarkers and/or staining protocols used in the present study also produced “non-specific staining” even in naïve mice. In addition to obvious astrocytic staining as shown previously (Xiong et al., 2023), FJC typically stained ependymal cells and pia mater (Supplementary Figure 1A). At higher magnification, basal bodies of ependymal cells appeared as patches of puncta (Supplementary Figure 1B), similar to what we showed previously (Xiong et al., 2014). Fibrous staining was prominent in pia mater wrapping the whole brain (Supplementary Figure 1C) and extending into the parenchyma as perivascular space (PVS), or Virchow–Robin spaces. Direct staining with Avidin-HRP (i.e., no primary and secondary antibodies applied) revealed a diffuse distribution pattern of endogenous biotin in the brain (Supplementary Figure 1D–F), including the cortex, hippocampus and white matter bundles where TBI-induced neuropathology has been detected (Xiong et al., 2023). Some of the avidin-stained puncta were arranged in rows in the white matter bundles (Supplementary Figure 1E, *arrowheads*), similar to the varicosities revealed in TBI patients with immunoperoxidase-ABC staining using 22C11 (Johnson et al., 2013). NeuroSilver could show dark staining of oligodendrocytes in white matter bundles in healthy brains (Supplementary Figure 1G, *inset*), with more oligodendrocyte staining after lFPI as reported previously (Xiong et al., 2023). Beaded fibrous staining could be clearly identified in pia mater (Supplementary Figure 1H, *inset*) and along perivascular spaces (Supplementary Figure 1I, *inset*) after NeuroSilver staining. NeuroSilver staining could also show irregular puncta along ventricular banks (Supplementary Figure 1I, *inset*). It should be remembered that such non-specific staining is identifiable in both control and injured brains, along with basal and/or pathological staining as described above.

## Discussion

In the present study we identified TBI-induced pathology in neuronal structures including cell bodies, dendrites and axons, which were positive to one or more of the three major TBI biomarkers: Fluoro-Jade C, the APP antibody Y188 and NeuroSilver. We also showed large patches of puncta in white matter bundles, which are likely to be of oligodendrocyte origin (Marklund, 2023; Xiong et al., 2023). The origin of the perivascular structures remains to be studied. We established that no single marker could visualize all these types of pathology effectively and over all relevant time points (Table 1).

Along with the traditional staining pattern previously reported, we found additional targets for these biomarkers. FJC, for instance, stains neuronal cell bodies (Yang et al., 2015, 2020), but can also detect beaded deformation of dendrites and Wallerian degeneration of axons as demonstrated here. Y188 clearly reveals axonal blebs in gray matter areas (or “axonal truncations” as previously described; Stone et al., 2000, with a different APP antibody), but also shows patches of punctate staining in white matter bundles. In addition, Y188 labels condensed neuronal cell bodies at later time points. NeuroSilver has been widely used to show neuronal cell body damage, and Wallerian degeneration together with axonal bulbs (Koliatsos et al., 2011; Xiong et al., 2023). In addition to these well-established patterns of staining, NeuroSilver can also detect axonal blebs (Xiong et al., 2023) and beaded deformation of dendrites.

The present study also highlights differences in the target specificity and/or time course of labeling for the respective markers, and identifies regional and animal model-dependent differences in labeling as well. For instance, while all three biomarkers labeled neuronal cell bodies after TBI, each marker showed cell body staining in a specific time window (Table 1), suggesting that these markers are identifying different components of the post-injury process. With regard to cell body staining, it is interesting to note that we also found regional specificity in the staining, as none of the three markers stained damaged, dying or dead cell bodies and beaded dendrites in CA1 with our non-penetrating lFPI injury model, even though nearby dentate gyrus and area CA3 were routinely stained (Xiong et al., 2023). By contrast, our preliminary experiments showed that the controlled cortical impact model of injury does produce abundant CA1 cell body staining (data not shown), suggesting injury protocol as yet another dimension of variability between the markers, and pointing perhaps to differences in the respective mechanisms of injury.

Although no conclusive evidence exists for the hypothesis that FJC detects dead or dying neurons after TBI (as opposed to simply damaged neurons), our results suggest that FJC staining does nonetheless detect early damage to neurons. Therefore, we use FJC staining as an early marker for brain injury, and perform immunostaining and/or silver staining to confirm dead or dying neurons (Xiong et al., 2023). By contrast, NeuroSilver and Y188-stained neurons were routinely associated with indices of neuronal death, and not simply neuronal damage. Cell debris, for instance, was consistently encountered among silver-stained neuronal cell bodies, suggesting the beginning of cellular deposition. Likewise, although slightly later in TBI process, Y188 could be used to show the last stage of neuronal death, based on the condensation of stained cell bodies and proximal dendrites (Xiong et al., 2023). At the early phase of the post-injury time range, FJC detects beaded deformation of dendrites as early as 6 hr after lFPI, whereas Neurosilver staining of this type of pathology does not emerge until 18 hr, making FJC a better choice for detecting early changes of this subcellular domain. On the other hand, NeuroSilver kit exposed much higher sensitivity at detecting Wallerian degeneration and axonal bulbs, than did FJC. Y188 clearly shows a connecting segment between the axonal bleb and its parent cell body, which is missing when stained with NeuroSilver kit. This discrepancy between NeuroSilver kit and Y188 may imply a target binding domain preference. In summary, there is no “one size fits all” marker for TBI. The utility of a given marker depends on many factors, and determining the “best” marker for a TBI study depends on making the best match possible between the sensitivity of the marker and the injury phenomenon of interest. Alternatively, and/or in the absence of a particular post-injury target, a combination of markers might best be used to ensure no type of pathology is missed.

A counter example for the benefit of using a combination of markers, comes from earlier TBI studies using the anti-APP antibody 22C11. Such studies often utilized a sole biomarker (22C11) to detect TBI neuropathology (Gentleman et al., 1993, 1995; Sherriff et al., 1994a,b; Graham et al., 2004; Reichard et al., 2005; Hortobágyi et al., 2007; Johnson et al., 2013; Ryu et al., 2014; Koch et al., 2020). Immunoperoxidase-ABC staining with 22C11 had been even recommended as the “Gold standard” for detecting axonal degeneration in TBI patients (Johnson et al., 2013, 2016), even though it was subsequently shown to be nonspecific when used for immunohistochemistry (Guo et al., 2012; Del Turco et al., 2016; Xiong et al., 2023), and problematically corrupted by endogenous biotin staining when using the ABC kit (Xiong et al., 2023). Such confusion might have been avoided if multiple markers had been used. We demonstrate that no single biomarker can serve as a TBI standard, based on the differences in temporal, structural and regional sensitivity. Instead, a combination of different biomarkers should be used and multiple time points need to be checked in order to get a comprehensive profile of TBI-induced neuropathology (Xiong et al., 2023). This strategy will be especially important in human studies evaluating injured brains, which are often collected over a wide range of time points after TBI.

When interpreting TBI data, “non-specific staining” (Table 1) should also be taken into account. Thus far, no studies have raised this point clearly. If immunoperoxidase-ABC is performed, the interference of endogenous biotin needs to be excluded, especially when the specificity of the primary antibody may be in doubt, such as for 22C11 (Guo et al., 2012; Del Turco et al., 2016; Xiong et al., 2023). By contrast, the staining patterns for Y188 are virtually identical for the immunoperoxidase versus immunofluorescent protocols (Xiong et al., 2023, and present study) because the primary antibody Y188 is specific and dominates the staining. Nonspecific silver staining within brain parenchyma was the most prominent in highly foliated structures such as the cerebellum, especially in its transverse slices from control and injured mice.

Several limitations exist in the current study. (1) The space constraints of a Brief Communication did not allow us to include sham figures. Since sham surgery can also produce damage, we consider naïve tissue to be a better comparison to injured for the purpose of assessing whether a given marker can be used to identify damaged tissue. As such, we focused our figures on injured tissue versus naïve tissue. Readers are encouraged to check our previous paper (Xiong et al., 2023) for IHC images in sham mice. (2) Staining of human tissue was not included. We are exploring the possibility of acquiring TBI patient samples for further investigation, but the added difficulty of obtaining and working with human tissue is beyond the scope of the current study. We note nonetheless that Y188 should be suitable for detecting neuropathology in human brains after TBI, since human APP is homologous to mouse and rat APP. (3) We used only male mice in the current study. We expect that all three biomarkers would work equally well for detecting neuropathology in tissue from both male and female animals after TBI as we are not aware of any sex-related differences in the pathological structures. We are fully aware that female hormones and menstrual status may affect TBI progress, and this is an important area of TBI research, but the primary goal of the present study was to evaluate the ability of these biomarkers to detect TBI-induced pathological structures. (4) Righting time and FJC staining were the primary measures used to confirm successful induction of an injury – mortality rate and lesion volume were not quantified, and behavior tests were not done. We record righting time as an initial index for evaluating injury severity, and mice injured with a 2.6 atm impact typically show a righting time of 5–15 min, which is much longer than the 1.5–5 min righting times for 1.8 atm injuries, or the 0.5−1.5 min range typical for sham injuries (craniectomy and isoflurane, but no pendulum drop). Positive FJC staining among cortical and hippocampal neurons was also used to confirm injury. (5) Careful quantitation of the staining was not performed. This brief report is a qualitative analysis, focusing on establishing biomarkers for detecting neuropathology after TBI. By and large the pathology examined here was either present or not present, and our results are presented in this context. In future studies, quantitative analysis could be performed between different subjects or models, using a specific marker (or combination of markers), to gauge the relative prevalence of the pathological structures observed.

Careful assessment of the underlying pathology is the cornerstone of our efforts to understand, treat and cure TBI. Using multiple markers and multiple time points, and being alert to the confounding effects of nonspecific staining, holds great promise for such work.

## Data availability statement

The original contributions presented in this study are included in this article/Supplementary material, further inquiries can be directed to the corresponding author.

## Ethics statement

The animal study was approved by the Institutional Animal Care and Use Committees of Children’s Hospital of Philadelphia and University of Pennsylvania. The study was conducted in accordance with the local legislation and institutional requirements.

## Author contributions

GX: Data curation, Formal analysis, Investigation, Methodology, Validation, Visualization, Writing−original draft. IJ: Investigation, Writing−review and editing. AF: Investigation, Writing−review and editing. HM: Investigation, Writing−review and editing. BJ: Validation, Writing−original draft. NC: Resources, Writing−review and editing. AC: Funding acquisition, Writing−review and editing.

## Supplementary material

Supplementary Figure 1 “Non-specific staining” in naïve mice by the three biomarkers of interest. **(A)** FJC staining in ependymal cells **(b)** and pia mater **(c)**. Heavily stained basal bodies of ependymal cells **(B)** and pial fibers **(C)** highlighted. **(D–F)** Endogenous biotin was detected in the external capsule **(e)** and dentate gyrus **(f)** by direct staining with Avidin-HRP. **(E)** Heterogenous punctate staining in the white matter bundle, with some arranged in rows (*arrows*). **(F)** Avidin staining predominantly seen in subgranular zone of the dentate gyrus. **(G–J)** Obvious staining with NeuroSilver kit. Intensely stained oligodendrocytes in external capsule **(G)**, beaded fibers in pia mater **(H)** and perivascular space **(I)**, or irregular puncta along ventricular bank **(J)**, highlighted in (***insets***), respectively. Scale bars: 50 μm in panels **(A,G–I)**; 10 μm in **(B,C)**; 5 μm in (*insets*).
