# Supplementary figures and images for: Temporal and structural sensitivities of major biomarkers for detecting neuropathology after traumatic brain injury in the mouse
Source: Front Neurosci. 2024 Jan 30;18:1339262. doi: 10.3389/fnins.2024.1339262 (PMC10865493; doi:10.3389/fnins.2024.1339262)

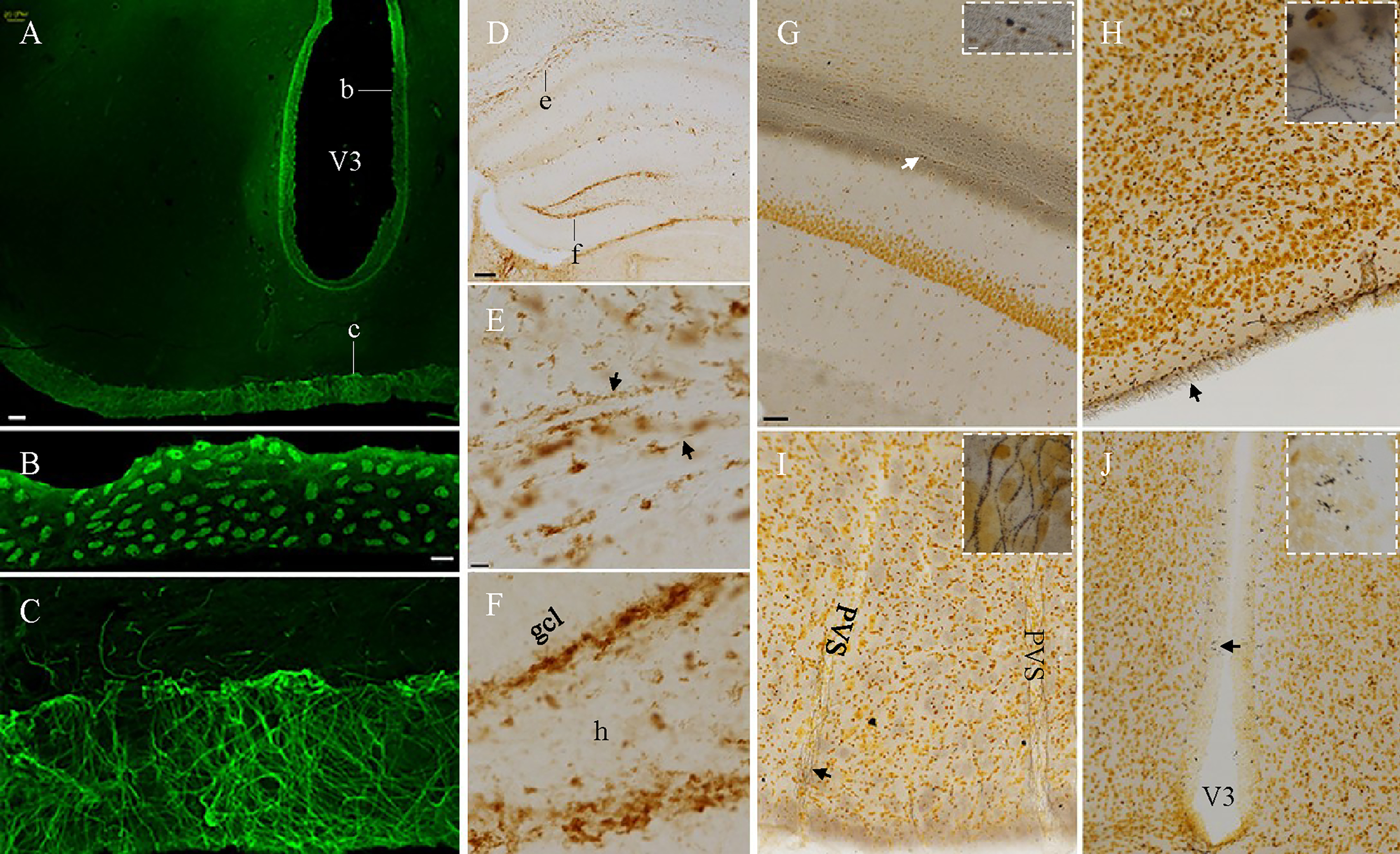

Supplement: Supplementary Figure 1 — “Non-specific staining” in naïve mice by the three biomarkers of interest. (A) FJC staining in ependymal cells (b) and pia mater (c). Heavily stained basal bodies of ependymal cells (B) and pial fibers (C) highlighted. (D–F) Endogenous biotin was detected in the external capsule (e) and dentate gyrus (f) by direct staining with Avidin-HRP. (E) Heterogenous punctate staining in the white matter bundle, with some arranged in rows (arrows). (F) Avidin staining predominantly seen in subgranular zone of the dentate gyrus. (G–J) Obvious staining with NeuroSilver kit. Intensely stained oligodendrocytes in external capsule (G), beaded fibers in pia mater (H) and perivascular space (I), or irregular puncta along ventricular bank (J), highlighted in (insets), respectively. Scale bars: 50 μm in panels (A,G–I); 10 μm in (B,C); 5 μm in (insets). [file Image_1.TIF]
